# Live Pups from Evaporatively Dried Mouse Sperm Stored at Ambient Temperature for up to 2 Years

**DOI:** 10.1371/journal.pone.0099809

**Published:** 2014-06-12

**Authors:** Jie Liu, Gloria Y. Lee, Joel A. Lawitts, Mehmet Toner, John D. Biggers

**Affiliations:** 1 Massachusetts General Hospital, Harvard Medical School and Shriners Hospital for Children, Boston, Massachusetts, United States of America; 2 Beth Israel Deaconess Medical Center, Boston, Massachusetts, United States of America; 3 Department of Cell Biology, Harvard Medical School, Boston, Massachusetts, United States of America; Clermont-Ferrand Univ·, France

## Abstract

The purpose of this study is to develop a mouse sperm preservation method based on evaporative drying. Mouse sperm were evaporatively dried and stored at 4°C and ambient temperature for 3 months to 2 years. Upon rehydration, a single sperm was injected into a mature oocyte to develop into a blastocyst after culture or a live birth after embryo transfer to a recipient female. For the samples stored at 4°C for 3, 6, 12, 18, and 24 months, the blastocyst formation rate was 61.5%, 49.1%, 31.5%, 32.2%, and 41.4%, respectively. The blastocyst rate for those stored at ambient temperature (∼22°C) for 3, 6, 12, and 18 months was 57.8%, 36.2%, 33.6%, and 34.4%, respectively. Fifteen, eight and three live pups were produced from sperm stored at room temperature for 12, 18, and 24 months, respectively. This is the first report of live offspring produced from dried mouse sperm stored at ambient temperature for up to 2 years. Based on these results, we suggest that evaporative drying is a potentially useful method for the routine preservation of mouse sperm.

## Introduction

With the fast advances in molecular genetics, genome sequencing, and reproductive technology, thousands of genetically engineered and mutant mouse strains have been used for studying disease processes and embryonic development. It is critical for economic reasons to preserve these valuable genotypes efficiently and reliably without the need of expensive breeding colonies. The banking of spermatozoa is emerging as the most efficient and cost effective approach. Sperm drying offers an attractive alternative to the conventional method of cryopreservation for mouse sperm because it enables storage at ambient temperatures. Two methods of drying are possible: evaporative drying and freeze drying. Evaporative drying, based on anhydrobiotic processes found in nature, is a simple, efficient, and cost effective process that does not require sophisticated equipment and can be easily implemented [1], [2]. Evaporative drying does not include freezing, an extra step that can cause harm. In contrast, freeze-drying is a multiple step procedure that requires sophisticated equipments and hours to accomplish [3].

Preservation of mouse sperm at ambient temperature has been challenging, with 3 months being the longest time reported that sperm stored at room temperature could generate blastocysts and/or live offspring upon injection into mouse oocytes [2], [4]–[6]. We herein demonstrate that with an improved evaporative drying method, using trehalose as a protective agent, blastocysts and offspring can be obtained from mouse sperm preserved at room temperature for up to 2 years.

## Materials and Methods

### Ethics Statement

All procedures involving animals were reviewed and approved by the Massachusetts General Hospital Subcommittee on Research Animal Care (no. A3596-01). All efforts were made to minimize animal suffering.

### Animals

One hundred and sixty-seven 2 to 4 months old B6C3F1 female mice (Jackson Laboratory, Bar Harbor, ME) were used as oocyte donors. Twenty-five 3 to 9 months old B6D2F1 male mice (Jackson Laboratory, Bar Harbor, ME) were sperm donors. Only the sperm from males mated in a prior week was used. ICR males and females (Taconic Farms, Germantown, NY) were used to produce vasectomized males and pseudopregnant females for embryo transfer.

### Reagents and Media

Trehalose was purchased from Ferro Pfanstiehl (Waukegan, IL). KSOM^aa^ Evolve w/HEPES and KSOM^aa^ Evolve media, containing glycyl-glutamine instead of glutamine, were provided by Zenith Biotech (Guilford, CT). The KSOM culture medium was KSOM^aa^ Evolve medium supplemented with 1 mg/ml bovine serum albumin. The EGTA (ethylene glycol tetraacetic acid) solution was 10 mM Tris-HCl buffer supplemented with 50 mM each of NaCl and EGTA with pH adjusted to 8.2–8.4 [7]. All other reagents were purchased from Sigma-Aldrich (St. Louis, MO) unless otherwise stated.

### Sperm Sample Preparation

Male mice were anesthetized with Isoflurane (Abbott laboratories, Chicago, IL) and euthanized by cervical dislocation. The caudal epididymides were removed, placed in 1 ml pre-warmed EGTA solution in a 60-mm center well culture dish (Corning Incorporated, Corning, NY), and cut in several places with a sterile needle to release the sperm. The EGTA solution containing the sperm was then transferred to a 1.5-ml centrifuge tube, which was held at room temperature for 10 min for swim-up. The top 100 µl of the sperm suspension was mixed with 100 µl α-hemolysin stock solution (0.025 mg/ml), and the mixture was incubated at room temperature for 30 min for sperm membrane poration. The porated sperm suspension was then mixed with 200 µl of the EGTA solution containing 200, 400, 600, 800, or 1000 mM trehalose to give a final trehalose concentration of 100, 200, 300, 400, or 500 mM. The mixtures were held at room temperature for 15 min before sonication. Sonication was performed as described [2] to separate sperm heads and tails.

### Optimal Concentration of Trehalose

**Figure 1:**
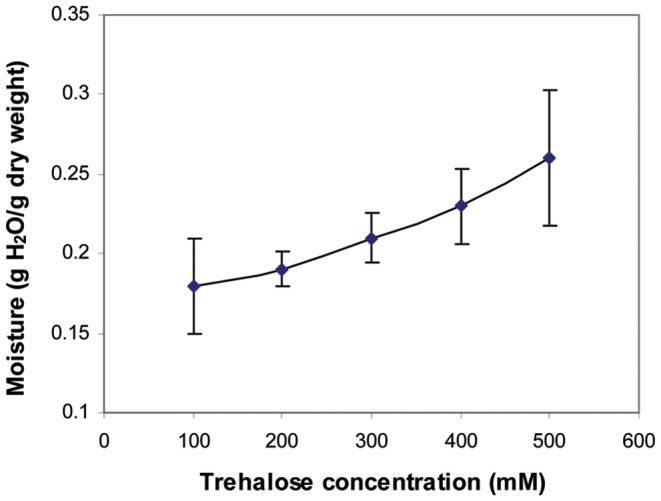
Moisture content and trehalose concentration. Moisture content of the samples was positively correlated with the concentration of trehalose. Error bars on Y axis indicate standard deviations.

A series of preliminary tests were performed to determine the optimum concentration of trehalose for evaporative drying and storage of mouse sperm. An evaporative drying procedure described previously was applied [2]. Briefly, each 20 µl sperm sample with 100, 200, 300, 400, or 500 mM trehalose was evenly spread on the 10-mm diameter etched ring in the center of a 25 mm×25 mm square glass slide, dried by a stream of nitrogen gas at 10 l/min for 6 min, and baked in an oven at 85°C for 6–7 days to determine the effect of trehalose concentration on moisture content. Either six or eight slides were dried for each treatment and the average moisture content was calculated. Slides were weighed before and after evaporative drying, and after baking on an ultra microbalance (Mettler Toledo Inc., Columbus, OH). Moisture content was calculated as follows [6]: Moisture content (g H_2_O/g dry weight)  =  (dried weight – baked weight)/baked weight. When the moisture content was above ∼0.2 g H_2_O/g dry weight, sperm samples had a high ability to ensure blastocyst development [6]. The moisture contents fell into this range when 300 mM trehalose was used (Figure 1); we then chose 300 mM trehalose for the following experiments.

### Evaporative Drying and Storing with Optimum Concentration of Trehalose

Samples with 300 mM trehalose were dried using the same procedure described above and stored in LiCl sorption jars [2] at 4°C and ambient temperature until the desired time point. Two to three samples from two to three different males each were stored for each storage time and temperature.

### Oocyte Preparation

Superovulation was induced in B6C3F1 females by two intraperitoneal injections of 0.15 ml PG 600 (Intervet Inc., Millsboro, DE) per mouse per injection, 48 h apart. Oocytes were retrieved 14 to 15 h after the second injection. Cumulus cells were removed by incubating the cumulus-oocyte complexes in 0.3 mg/ml hyaluronidase in KSOM^aa^ Evolve w/HEPES medium at room temperature for 2 min and brief pipetting. Denuded oocytes were washed and incubated in the KSOM culture medium at 37°C with 6% CO_2_ in humidified air until use.

### Intracytoplasmic Sperm Injection (ICSI)

ICSI was applied using a modified conventional method [8]. Twenty micro liters of sterile distilled water were added to each dried sample (a thin film) to rehydrate to its original volume. The suspension was mixed, transferred to a micro-centrifuge tube, which was kept at 4°C during experiments. Two to three micro liters of the sperm suspension were mixed with 15 µl 12% polyvinyl pyrrolidone (PVP) in H_2_O. A randomly selected sperm head was picked up by an injection pipette and inserted into an oocyte. A group of 15 to 20 oocytes were injected before a new PVP-sperm drop was prepared. Injected oocytes were washed and cultured in the KSOM culture medium at 37°C with 6% CO_2_ in humidified air.

### Embryo Culture

Between 5 and 10 injected oocytes were placed in each 50 µl drop of the KSOM culture medium [9], covered with mineral oil, and cultured at 37°C with 6% CO_2_ in humidified air. The developmental progress of the embryos was recorded daily for 6 days.

### Embryo Transfer

Two- to four-cell embryos were transferred to pseudopregnant ICR females, which had been mated with vasectomized males. The number of embryos transferred on a given day depended on the number of two- to four-cell embryos and the number of recipients available. Embryos were allowed to develop to term. Pups were kept up to 4 weeks of age.

### Statistical Methods

**Figure 2:**
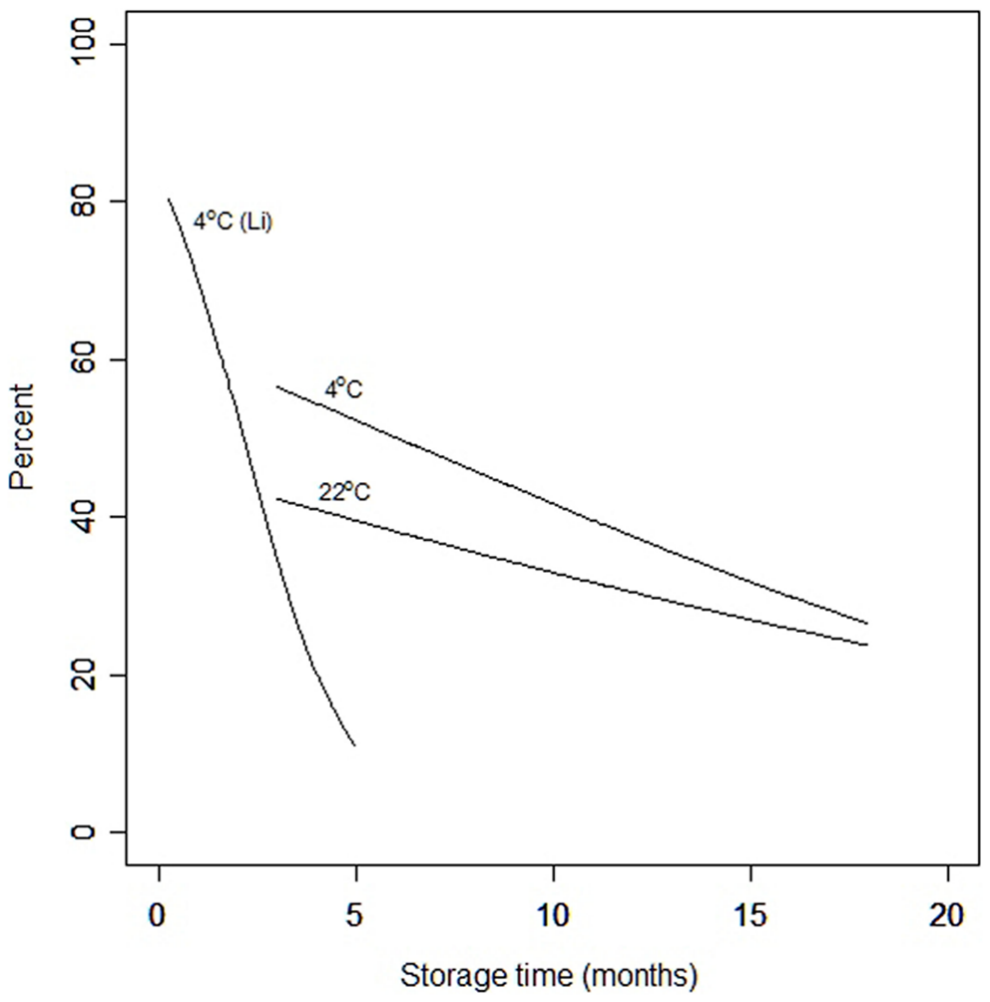
Logistic regressions listed in **Table 3** after transformation to their percentage scale.

**Figure 3:**
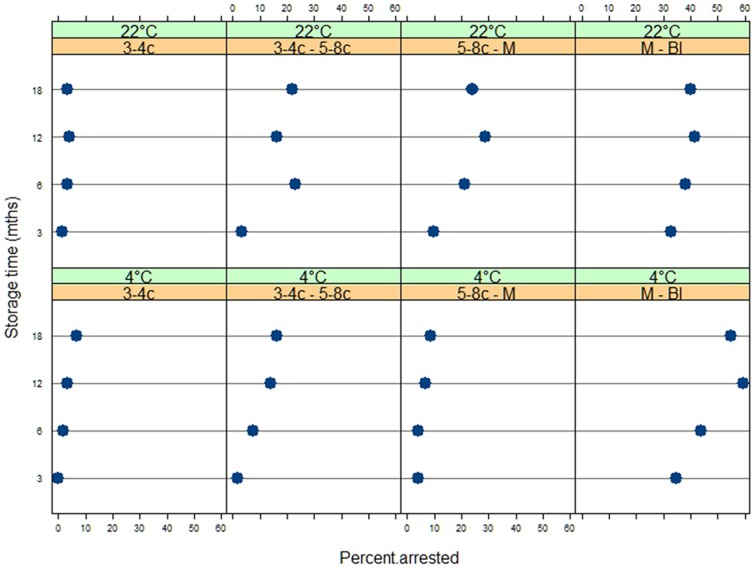
Trellis graph of dotplots. Each row corresponds to a storage temperature and each column to a developmental transition. It shows the effect of storage time on the percent of arrested embryos conditioned on stage (developmental transition point) and temperature. The stages, across the columns of graphs, are: 3/4 cells, 3/4–5/8 cells, 5/8 cells – morula, morula – blastocysts. Percent arrested: percent of embryos that arrested at the next stage of development.

Unordered, singly ordered and doubly ordered contingency tables were analyzed using the exact Fisher, Kruskal-Wallis, and Jonckheere-Terpstra tests of significance, respectively. The analyses were done using the StatXact 10 Package (Cytel Inc., Cambridge, MA). Logistic regression analysis was done using the Logistic 10 Package (Cytel Inc., Cambridge, MA). Figures 2 and 3 were prepared using S-Plus 6 (Insightful Corporation, Seattle, WA). Differences were considered significant at P<0.05.

## Results

### In vitro Development of Embryos Produced from Evaporatively Dried Sperm

#### Fertilization

The numbers and percentages of two-cell embryos that developed after the injection into oocytes of evaporatively dried sperm, stored at either 4°C or ambient temperature, are shown in Table 1. The percentages were assumed to estimate the fertilization rates. The fertilization rates using sperm stored for 3, 6, 12 and 18 months at 4°C were not significantly different (P = 0.077), whereas those stored at ambient temperature were different (P = 0.028). These differences are due to a small depression of the fertilization rates using the sperm stored for 18 months. Overall 88.5% and 87.6% of oocytes were fertilized at 4°C and ambient temperature, respectively.

**Table 1:** The number of two-cell embryos (fertilization rate) that developed from mouse ova injected with a mouse sperm stored for different times and temperature using ICSI.

Storage time (mon)	Storage temperature	
	4°C	ambient temperature
3	52/57 (91.2%)	64/69 (92.8%)
6	55/60 (91.7%)	58/64 (90.6%)
12	89/99 (89.9%)	122/140 (87.1%)
18	59/72 (81.9%)	61/75 (81.3%)
Total	255/288 (88.5%)	305/348 (87.6%)
Exact Kruskall-Wallis test	P = 0.077	P = 0.028

The fertilization rate from freshly isolated sperm heads without storage (fresh control) was 90.7% (78/86).

#### Development

The distributions of the developmental stages following ICSI reached on Day 5 after different storage times at either 4°C or ambient temperature and not stored (fresh control) are listed in Table 2 (Day 0 is defined as the day ICSI was done). The sets of distributions observed for 4°C and ambient temperature are significantly different (4°C, P = 0.0011; 22°C, P = 0.002). Almost all injected ova underwent some development including those stored for 18 months. The rates of blastocyst formation in the group stored at 4°C fell from 61.5% after 3 months to 32.2% after 18 months. The rates of blastocyst formation in the group stored at ambient temperature fell from 57.8% after 3 months to 34.4% after 18 months. A considerable number of embryos developed only to the morula stage at both 4°C and ambient temperature, but unlike those that developed to the blastocyst stage, the rates of morula formation were unaffected by the duration of storage. The overall rates of morula development were 40% and 24.3% at 4°C and ambient temperature, respectively. Mature oocytes penetrated by an injection needle without inserting a sperm (sham control) remained one cell before fragmenting or degenerating (unpublished data).

**Table 2:** The number of embryos that developed to different stages from mouse ova injected with a mouse sperm stored for different times and temperature using ICSI.

Storage temp.	Storage time (mon)	Developmental stage (5 days post-fertilization)					Total
		2-cell	3-4-cell	5-8-cell	Morula	Blastocyst	
4°C	3	0	1 (1.92%)	2 (3.85%)	17 (32.7%)	32 (61.5%)	52 (100%)
	6	1 (1.82%)	4 (7.27%)	2 (3.64%)	21 (38.2%)	27 (49.1%)	55 (100%)
	12	3 (3.37%)	12 (13.5%)	5 (5.62%)	41 (46.1%)	28 (31.5%)	89 (100%)
	18	4 (6.78%)	9 (15.3%)	4 (6.78%)	23 (39.0%)	19 (32.2%)	59 (100%)
22°C	3	1 (1.56%)	2 (3.13%)	6 (9.38%)	18 (28.1%)	37 (57.8%)	64 (100%)
	6	2 (3.45%)	13 (22.4%)	9 (15.5%)	13 (22.4%)	21 (36.2%)	58 (100%)
	12	5 (4.1%)	19 (15.6%)	28 (23.0%)	29 (23.8%)	41 (33.6%)	122 (100%)
	18	2 (3.28%)	13 (21.3%)	11 (18.0%)	14 (23.0%)	21 (34.4%)	61 (100%)
Fresh control	/	0	4 (5.1%)	2 (2.6%)	9 (11.5%)	63 (80.8%)	78 (100%)

Linear regressions were fitted to the logits of the proportions of blastocysts formed against storage time for each storage temperature, using the data shown in Table 2. The estimates of the parameters of the two regression lines and their 95% confidence limits are shown in Table 3. The regression lines obtained after transforming the logits to proportions are shown in Figure 2 which relates the percentages of blastocysts to storage time at 4°C and ambient temperature. Although the graphs suggest that more blastocysts developed when the sperm were stored at 4°C the differences between estimates of the parameters of the two lines are not significantly different. Thus loss of functionality of sperm is similar for both storage temperatures. Moreover, after storage at both temperatures, a significant percentage of sperm (20%–30%) remained functional after 18 months. For comparison, data from a previous paper [10] where sperm survived poorly at 4 °C were also included in the diagram.

**Table 3:** The estimated parameters and their confidence limits (P = 0.95) of the logistic regressions (b and a) fitted to the data from storage at 4 °C and ambient temperature.

Storage temperature	Parameter	
	b	a
4°C	-0.08546 (-0.1335 to - 0.0374)	0.5175 (-0.01901 to 1.054)
ambient temperature	-0.05713 (-0.1017 to - 0.01252)	0.1385 (-0.3577 to 0.6348)
4°C (previous study)*	-0.74	1.591

The table also includes the estimated parameters from a logistic regression fitted to the data from storage at 4°C reported in a previous paper [10].

*The regression coefficient was transformed to convert the independent variable from weeks to months.

The rates of failure to develop at each developmental stage can be calculated from the results in Table 2. These rates are summarized in the trellis graph shown in Figure 3 which consists of two rows of four panels. Each row corresponds to a storage temperature (4°C and ambient temperature), and each column to a developmental transition (3/4-cell, 3/4-5/8-cell, 5/8-cell-morula, morula-blastocyst). Each dotplot relates the number of embryos that become arrested at each stage as a percentage of embryos that had reached the previous stage. These trellis dotplots help identify patterns of developmental failure in the data.

#### Preliminary studies with sperm stored for 2 years

When evaporatively dried sperm stored at 4°C for 24 months were injected into mouse oocytes, 92.6% (87/94) developed to two-cell embryos. Of the 87 two-cell embryos, 36 (41.4%) became blastocysts by day 6. Due to the limited number of samples available at ambient temperature for 2 years, all of the 51 embryos produced from these sperm were transferred into female recipients.

### Embryo Transfer and Live Births

**Figure 4:**
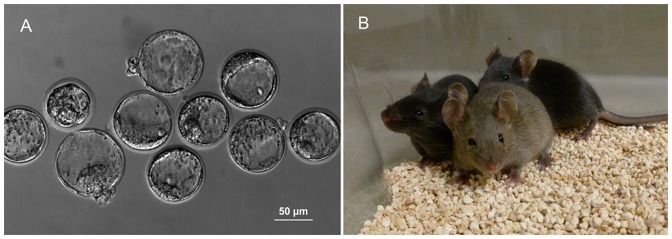
Blastocysts and mice produced from evaporatively dried sperm. A) Blastocysts from sperm stored at 4°C for 2 years. B) Mice produced from sperm stored at ambient temperature for 2 years.

One hundred and forty 2- to 4-cell embryos produced from sperm stored at ambient temperature for 12 months were transferred to three pseudopregnant females and a total of 15 live pups were born (10.7%). The transfer of 86 two- to four-cell embryos from sperm stored at ambient temperature for 18 months to two pseudopregnant females produced 8 live pups (9.3%). When 51 one- to two-cell embryos from sperm stored at ambient temperature for 2 years were transferred to two pseudopregnant females, three live pups were born (5.9%). For controls, 32 two- to four-cell embryos produced from freshly isolated sperm heads were transferred into two females. Eleven pups were born (34.4%) (Table 4). All of the pups looked healthy at 4 weeks of age (Figure 4).

**Table 4:** The embryo transfer results for evaporatively dried sperm stored at ambient temperature for 12 to 24 months.

Storage time	embryos transferred	No. pups (%)
12 months	140 two- to four-cells	15 (10.7)
18 months	86 two- to four-cells	8 (9.3)
24 months	51 one- to two-cells	3 (5.9)
Fresh control	32 two- to four-cells	11 (34.4)

## Discussion

The results reported in this paper demonstrate that evaporatively dried mouse sperm can be stored at ambient temperature for up to 2 years. After rehydration, ICSI can be used to inseminate mature ova with the hydrated sperm, and the resulting embryos transferred to surrogate mothers to produce live offspring.

Very high percentages of two-cell embryos developed from ova injected with evaporatively dried sperm when stored at 4°C and ambient temperature for 3 months to 2 years, and a significant number of them developed into blastocysts. This cannot occur unless the ova are activated as soon as a sperm head is injected, possibly by a 70 kDa protein (phospholipase C zeta [PLCζ]) [11]. Thus the activity of this protein is not destroyed by evaporative drying and long term storage.

Less than 7 and 4 percent of the 2-cell stage embryos arrested using evaporatively dried sperm stored up to 18 months at 4°C and ambient temperature, respectively. These low losses are expected since the introduced male genome does not become functional in the mouse until the maternal zygotic transition is completed at the 3-4-cell stage [12], [13]. Low arrests of embryos occurred at the 3/4-5/8-transition (<17%) and the 5/8-morula transition (<9%) when evaporatively dried sperm stored at 4°C were used. In contrast many more embryos arrested at the morula-blastocyst transition (<55%). The numbers of arrested embryos observed using sperm stored at ambient temperature are larger and more variable, but follow a similar pattern (3/4-5/8-transition, <23%; 5/8-morula transition, <24%; morula-blastocyst transition <40%). In general the longer the storage time at 4°C and ambient temperature, the greater the numbers of arrested embryos. There is now extensive evidence that preimplanation development is regulated throughout by a complex genetic program [14]–[16]. In addition to the maternal zygotic transition the morula blastocyst transition is a critical time when the first tightly sealed epithelium develops (the trophoblast) which is capable of controlling the milieu intérieur of the embryo [17]. Possibly damage to the sperm DNA during drying, storing and hydration, which does not become active until after the maternal zygotic transition, leads to the loss of developmental potential in some of the embryos.

The survival of sperm stored for 3 months and longer using our present technique is far greater than results published earlier, also shown in Figure 2, with a different technique [10]. In our previous protocol, 500 mM trehalose was used instead of 300 mM. Also, after drying, instead of storing in the LiCl sorption jars, each slide was vacuum-sealed in a plastic bag which was then vacuum-packed into a Mylar foil bag. We now know that the double vacuum-sealed package is not moisture proof and the samples stored in it lose water over time [6]. Although a lower trehalose concentration might help improve the long term survival of evaporatively dried sperm, we believe the low humidity LiCl sorption jars with minimal moisture fluctuation [2] contributed the most to the much improved results. This may suggest that minute amounts of water are required for the survival of biomaterials like sperm. Molecular mobility is reversely correlated with storage stability. With decreasing water content, molecular mobility reaches a minimum, and increases again at very low water content, implying that too dry and too humid conditions increase mobility and reduce longevity [18]. Finding the optimal moisture content and humidity level for storage may further increase shelf life for dried sperm samples.

The mouse sperm head consists almost entirely of a nucleus with very little cytoplasm. α-hemolysin was used to porate the mouse sperm membrane to facilitate the entry of trehalose. After entering the cell, trehalose is able to penetrate into the sperm nucleus [19] and contribute to protecting the DNA [20] and other molecules inside. A densely packed nucleus, inclusion of trehalose in the drying solution, and the facilitated entry of trehalose into the sperm cell have all contributed to the long term preservation of mouse sperm at room temperature.

Freeze drying is well known for dry storage of biomaterials including sperm. Although freeze-dried sperm can be stored long term at 4°C, the chromosomes of sperm stored at 24°C were considerably degraded after 3 months of storage [4], [21]. Attempts to generate offspring from sperm freeze-dried and stored at ambient temperature for 5 months [4] and one year [22] were unsuccessful. Other methods involving vacuum dried [23] and heat dried [24] sperm were able to produce blastocysts after a short period of storage at 25°C (7–10 days), but the rates were low (∼5%). The results we report here with evaporative drying of mouse sperm are the longest storage at ambient temperature for blastocyst development after ICSI and offspring after embryo transfer. We propose that evaporative drying is potentially valuable for the storage of mouse sperm obtained from valuable genotypes. Further work should focus on the normality of the sperm heads that survive long term storage and of the offspring produced by the technique.
